# Complex Role of Microbiome in Pancreatic Tumorigenesis: Potential Therapeutic Implications

**DOI:** 10.3390/cells11121900

**Published:** 2022-06-11

**Authors:** Suneetha Amara, Li V. Yang, Venkataswarup Tiriveedhi, Mahvish Muzaffar

**Affiliations:** 1 Division of Hematology/Oncology, Department of Internal Medicine, Brody School of Medicine, East Carolina University, Greenville, NC 27834, USA; yangl@ecu.edu (L.V.Y.); muzaffarm@ecu.edu (M.M.); 2 Department of Biological Sciences, Tennessee State University, Nashville, TN 37209, USA; vtirivee@tnstate.edu; 3 Department of Pharmacology, Vanderbilt University, Nashville, TN 37212, USA

**Keywords:** pancreatic cancer, immunotherapy, microbiome, metabolites, inflammation

## Abstract

Pancreatic cancer (PC) is the fourth leading cause of cancer-related mortality with limited diagnostic and therapeutic options. Although immunotherapy has shown promise in the treatment of several cancers, its role in pancreatic cancer is rather limited. Several studies have focused on determining the role of the tumor microenvironment with cancer-cell-intrinsic events and tumor-infiltrating immune cellular properties. However, in the past decade, there has been emerging research aimed at delineating the role of the host microbiome, including the metabolites from microbes and host responses, on pancreatic tumorigenesis. Importantly, there is emerging evidence suggesting the beneficial role of a gut microbiome transplant to improve immunotherapeutic outcomes in cancer patients. In this review, we summarize the recent understanding of the role of the microbiome in pancreatic cancer progression, along with its clinical diagnostic and therapeutic implications.

## 1. Introduction

Pancreatic cancer (PC) is the fourth leading cause of cancer-related deaths in the USA [1]. An estimated 62,210 new cases of PC are predicted to be diagnosed in 2022. While surgical resection remains the only curative therapy, only 10–20% of patients have resectable tumors at diagnosis [2]. Unfortunately, most of the patients at the time of diagnosis are at an advanced stage and with distant metastases, thus, limiting treatment options [3]. Pancreatic ductal adenocarcinoma (PDAC) accounts for more than 90% of all PCs. Despite continued efforts, the 5-year overall survival rate of PDAC patients remains at approximately 10% for all stages combined and deteriorates to 3% for those with distant metastasis [4]. Under extremely rare early detection instances, when the primary tumor is confined to the pancreas and is below 2 cm in diameter, the 5-year survival rate dramatically increases to 46% [5]. Hence, early screening, prevention and novel therapeutic approaches are needed for PC management.

The number of bacteria inhabiting the human body is estimated to be in the similar range as the total number of cells in the human body [6]. With advancements in sequencing technology, an in-depth examination and characterization of the genetic make-up of the microbiota (referred to as the microbiome) has become affordable. The human microbiome plays an essential role in maintaining body homeostasis, and an imbalance of the microbiota, a state known as dysbiosis, can contribute to the pathogenesis of many diseases [7]. In general, while healthy microbiota (eubiosis) contribute to a healthy immune system, a microbial dysbiosis is recognized to contribute to carcinogenesis and poor treatment outcomes [8]. Pathogenic microbes linked to human infections have also been identified as etiologic agents for 15%–20% of global cancer cases [9]. In this communication, we review the current understanding of the impact of the microbiome on PC tumorigenesis, along with its potential impact on therapeutic outcomes.

## 2. Pancreatic Microbiome

In general, while the pancreas was thought to be out of the reach of the gut microbiota, several studies have found the existence of bacteria in normal pancreatic tissue and pancreatic tumor samples. However, the challenge remains on defining the ‘normal’ microbiota for the pancreas. Various studies have found that while *Bacteroidetes* was found in pancreatic tissue from healthy samples, pancreatic cancer samples showed higher levels of three microbial taxa, namely, *Proteobacteria*, *Enterobacteriaceae*, and *Pseudomonadaceae*, of which *Bacteroidetes* and *Firmicutes* were the most common [10,11,12]. Along with bacteria, fungal colonization (mycobiome) with *Malassezia* species was enriched in PDAC samples [13]. Along with pancreatic localization, a differential gut and oral microbiota profile has been shown to correlate with pancreatic cancer prevalence. There was a significantly lower diversity (alpha diversity) of gut microbiota and mycobiota in pancreatic cancer patients, and were enriched with *Bacteroidetes*, *Klebsiella* and *Ascomycota*, along with lower concentrations of *Firmicutes* and *Proteobacteria* [14,15,16,17]. In contrast, an analysis of the oral microbiome demonstrated that pancreatic cancer patients demonstrated a higher diversity of microbiota with *Haemophilus*, *Porphyromonas*, *Leptotrichia* and *Fusobacteria* compared to healthy adults [18]. In another study, the presence of oral pathogens, *P. gingivalis* and *Aggregatibacter actinomycetemcomitans*, was associated with a higher risk of pancreatic cancer, while *Fusobacteria* was associated with a decreased risk [19]. The localization of various bacterial species with reported implications on PC are noted in Table 1.

In a study by Geller et al., 76% of the 113 PDAC tissue samples detected bacterial DNA, while only 15% (*p* < 0.005) of normal pancreas tissue had bacterial localization [29]. Murine studies with fluorescence-tagged micro/mycobiota demonstrated that within half-an-hour of oral administration, the tagged *E. faecalis*, *E coli* and *Saccharomyces cerevisiae* were found in mouse pancreatic tissue, suggesting that bacterial colonization to the pancreas can happen through the gastrointestinal route, possibly through the pancreatic duct [11,13]. However, in stark contrast, in another murine study, orally administered *Campylobacter jejuni* did not colonize in pancreatic tissue even after 8 weeks [30]. Interestingly, in an antibiotic-treated mouse model, non-invasive *Salmonella* was carried from the gut lumen to the mesenteric lymph nodes following antibiotic treatment with CX3CR1^+^ monocyte/dendritic cells [31]. The reasons for this opposing evidence need further study to determine if pancreatic colonization is microbiota-specific or host-specific. The differences in study sizes, designs, sampling methods and primers used for 16S rRNA amplification make interpretation and generalization difficult. Moreover, the decrease in gut microbial diversity in cancer patients compared to healthy individuals, along with the differences in oro-intestinal microbiome between pancreatitis and pancreatic cancer, makes the conclusions on cancer-specific microbiome difficult [32,33,34,35].

## 3. Role of Microbiome in Pancreatic Carcinogenesis

Several molecular mechanisms have been suggested to mediate microbial dysbiosis and pancreatic cancer. An innate-immune and pro-inflammatory signaling molecule, such as toll-like receptor (TLR)-2/4, was considered to play a role in *P. gingivalis*-mediated pancreatic carcinogenesis [36]. A metabolic influencing signaling factor, Akt, was also shown to play a role in dysbiosis-mediated carcinogenesis [37]. A genetically engineered murine tumor model (Kras^G12D^/PTEN^lox/+^) demonstrated that antibiotic treatment decreased the incidence of cancer [30]. Germ-free studies have shown that a lack of microbial growth was associated with increased anti-tumor M1 macrophage differentiation, tumor infiltration with cytotoxic CD8+ and CD4+ T cells, decreased myeloid-derived suppressor cells (MDSCs) and enhanced anti-tumor effects of PD-1 monoclonal antibody therapy [11,38]. Along these lines, another important observation was that prior antibiotic exposure, but not concurrent antibiotic use, can negatively impact the clinical efficacy of immunotherapy in some non-PDAC tumors [39]. These apparently contradictory literature evidences indicate the need for further molecular and functional microbial studies in PC [40,41].

### 3.1. Alterations of Microbial Metabolites

Microbial dysbiosis reduces the thickness of the mucus layer and, thus, decreases the host antimicrobial defense. This would also cause a decreased release of short-chain fatty acids (SCFAs) and gut peptides such as glucagon-like peptide-1 (GLP-1) and peptide YY [42]. The microbial fermentation of fiber produces SCFAs, which interact with host G-protein-coupled receptors, resulting in reduced chronic inflammation and the suppression of carcinogenesis [43,44]. The SCFAs have also shown to suppress histone deacetylase and induce apoptosis, leading to the inhibition of cell proliferation and metastasis [45,46]. However, in contrast, SCFAs have also shown to be correlated with the expansion of immunosuppressive pro-cancer regulatory CD4+T cells (Treg) and a resistance to anti-cancer immune-checkpoint inhibitor therapy [47]. This apparently conflicting role of SCFAs, with direct anti-tumor effects on cancer cells and pro-tumor immunosuppressive immune cells in the tumor microenvironment, only points out the need for a more thorough study to determine the role of microbial dysbiosis-mediated SCFA production on carcinogenesis.

The reduced thickness of the gut mucous membrane would cause the absorption of the released bacterial inflammatory metabolites, such as lipopolysaccharides (LPS) and lipoteichoic acid (LTA), into the host’s circulation. These bacterial metabolites, which are specifically released from Gram-positive bacteria, induce an inflammatory activation of TLR-mediated innate immune responses, along with the pro-inflammatory activation of macrophages, CD4+ and CD8+ T adaptive immune cells. Murine studies have demonstrated that these inflammatory events mediated by infection of *Enterococcus faecalis* could lead to chronic pancreatitis, an important predisposing factor for pancreatic carcinogenesis [48]. The 7α-dehydroxylating bacteria metabolize host cholic acid from bile to deoxycholic acid (DCA), which is associated with DNA damage and genome instability, potentially inducing PC tumorigenesis [49,50,51]. Further, DCA is known to upregulate the epidermal growth factor receptor (EGFR), mitogen-activated protein kinase (MAPK) and STAT3 oncogenic-signaling pathways [52]. Studies by Halimi et al. have shown that the isolation of the pancreatic microbiome obtained from pancreatic cystic lesions associated with invasive cancer demonstrated the presence of gamma-Proteobacteria and Bacilli. Interestingly, the ex vivo co-culture of this isolated pancreatic microbiota with an immortalized primary healthy human pancreatic cell line (hTERT-HPNE cells), early pancreatic adenocarcinoma cell line (Capan-2 cells) and late pancreatic adenocarcinoma cell line (AsPC-1 cells), demonstrated that the bacteria were able to enter all three cell lines and induce DNA double-strand damage. This ex vivo damage of pancreatic cells by the isolated pancreatic microbiota was inhibited by a co-treatment using the antibiotic gentamycin, suggesting a direct DNA-damaging impact of pancreatic microbiota with possible oncogenesis [53]. Similarly, butyrate (an SCFA) is considered to interfere with epigenetic modifications and transcriptional gene regulation [54]. Certain bacterial toxins, such as colibactin and cyclomodulins (specifically *Bacteroides fragilis* toxins), are known to trigger double-stranded DNA damage and interfere with cell cycle repair, leading to carcinogenesis [55,56].

### 3.2. Microbiota-Mediated Immunoregulation

Host immune cells interplay with gut microbes and maintain a symbiotic relationship to maintain human health [57]. Compositions of the microbiota are influenced by the immune system. Conversely, gut microbiota also plays an indispensable role in the maturation and continued education of the host immune system [58]. This delicate homeostatic balance, when disrupted, could lead to abnormal immune responses and tumorigenesis [59,60].

A limited number of germline-encoded pattern recognition receptors (PRRs) exist in the innate immune system. These receptors recognize the ‘non-host-self’ nature of microorganisms’ genetic material and proteins, often known as pathogen-associated molecular patterns. In addition to detecting conserved microbe-associated molecular patterns, TLRs can also be activated by inflammation or damage-associated molecular patterns (DAMPs). As downstream targets of these pattern-recognition molecules, the NF-κB and MAPK signaling pathways are activated, which initiate cytokine production and the further recruitment of pro-inflammatory entities that are ultimately involved in the development of cancer [61]. Importantly, one example of protection against pancreatic carcinogenesis is the blockade of the activation of TLR4/7 to inhibit its interactions with the STAT3, Notch, NF-κB and MAPK pathways. This effect has been successfully tested in systemic lupus erythematosus [62]. Similarly, several studies have focused on another PRR, the nucleotide-binding oligomerization domain (Nod)-like receptors (NLRs). These receptors (such as NLRP1, NLRP3 and NLRP4) recognize microbial signals to activate the caspase-1 inflammasome complex and induce the secretion of interleukin (IL)-1β and IL-18 [63]. IL-1β expression in a pancreatic tumor microenvironment is associated with treatment resistance and poor prognosis. The microbial dysbiosis could induce TLR4-NLRP3 signaling, resulting in an enhanced secretion of IL-1β by pancreatic tumor cells [64]. This secreted IL-1β, through the activation of pancreatic stellate cells, could induce mesenchymal fibrosis, which is shown to cause chemo/immuno-therapeutic resistance [64,65]. Additionally, NLRs participate in bacterial clearance by inducing the activation of NF-κB, P38/MAP kinase and interferon signaling, regulating autophagy-associated protein expressions and promoting autophagosome formation (such as NOD1 and NOD2) [66,67]. The intestinal microbiota also plays a critical role in the maturation and continued education of the host immune system [58], provides protection against pathogen overgrowth [68] and influences host cell proliferation [69] and vascularization [70].

**Figure 1:**
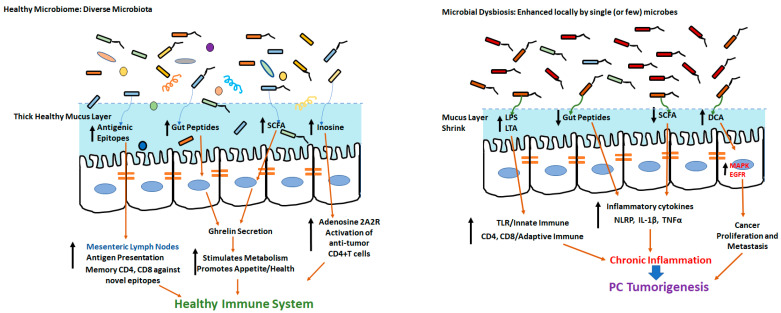
In the presence of healthy microbiome with diverse microbes, the gut epithelium has thick intact mucus membrane and promotes immune health through diverse antigenic epitopes and development of novel memory CD4 and CD8+T cells. Microbial dysbiosis and colonization of single (or a few) harmful microbes could lead to ablation of mucous membrane and release of deleterious metabolites leading to tumorigenesis.

Along with the modulation of the innate immune responses mentioned above, the pancreatic microbiome also promotes pro-cancerous adaptive immune responses. Using preclinical pancreatic cancer models, Pushalkar et al. demonstrated that the passive transfer of bacteria obtained from pancreatic ductal carcinoma hosts into normal mice induced oncogenesis. Further, the ablation of the pancreatic microbiome was associated with a reduction in the frequency of tumor-infiltrating immune cells with immunosuppressive tumorigenic myeloid-derived suppressor cells (MDSCs), along with an increase in anti-tumor M1 macrophage, Th1/CD4+T cells and CD8+T cells in the pancreatic tumor microenvironment. Additionally, the ablation of the microbiome caused an enhanced expression of PD1 on tumor-infiltrating CD4+ and CD8+ T cells, thus, enhancing the immunotherapeutic efficacy of anti-PD1 monoclonal antibodies (mAbs) [11]. Along similar lines, studies by Riquelme et al. demonstrated that long-term survivors (LTSs) of human pancreatic ductal adenocarcinoma had a higher microbial diversity than short-term survivors (STSs). This was also associated with a higher frequency of anti-tumor cytotoxic granzyme+CD8+T cells [71]. An overview of the microbiome-mediated modulation of innate and adaptive immune responses leading to potential carcinogenesis is shown in Figure 1.

## 4. Secondary Impact of Lifestyle Co-Morbidities on Microbiome-Modulated Carcinogenesis

Gut microbiota is also known to be impacted by lifestyle and metabolic co-morbidities, such as a high-fat/low-fiber diet, obesity, type 2 diabetes, smoking and alcohol consumption. Dysbiosis-mediated obesity could increase the risk of cancer due to the generation of pro-carcinogenic microbial metabolites, the modulation of gut peptides and pro-inflammatory tumorigenic changes [72,73]. Chronic pancreatitis is a strong risk factor for PC [74], presenting as a localized or systemic inflammation. Non-alcoholic fatty pancreatic disease was identified as a novel clinical obesity-related disease that increases pancreatic fatty degeneration and may progress to chronic inflammation and PC [75]. Bacterial overgrowth in the small intestine, as evidenced by a duodenal fluid analysis, was observed in 92% of chronic pancreatitis patients [76]. In contrast, a meta-analysis by El Kurdi et al. demonstrated that small intestinal bacterial overgrowth accounts for only 38% of chronic pancreatitis complications [77]. Similarly, in type 2 diabetic patients, an increase in *Lactobacillus* species along with a decrease in SCFA (butyrate)-producing *Roseburia intestinalis* and *Faecalibacterium prausnitzii* was observed [78]. These data warrant further study to understand the interplay between lifestyle-associated cancer co-morbidities and microbiota towards pancreatic carcinogenesis.

## 5. Microbiome-Specific Biomarkers

Unfortunately, there are only limited options for the clinical usage of a viable and effective diagnostic/prognostic biomarker for PC. Despite low sensitivity and specificity, the carbohydrate antigen 19–9 (CA19–9) continues to be the only available biomarker utilized in PC [79]. As several studies have shown an association between specific bacterial species and pancreatic cancer, an important area of research would be the identification of a novel microbial biomarker for pancreatic cancer. In a study with 283 PDAC tumor samples, *Fusobacterium* species were detected in 8.8% of the tumor samples and demonstrated a significant positive correlation along with a decreased cancer-survival rate, independent of other clinical and molecular features [21]. Similar evidence was found in a study using a duodenal fluid analysis, wherein *Fusobacterium* was correlated with short-term survivors (STSs < 5 years) of PDAC [17]. Another study by Riquelme et al. showed that microbial diversity, as a composite biomarker, was higher in long-term survivors (LTSs > 5 years). Further, the LTS cohort demonstrated microbial signatures enriched with *Pseudoxanthomonas*, *Streptomyces*, *Saccharopolyspora* and *Bacillus clausii* [71]. Farrell et al. used a qPCR-based technique to show that, compared to healthy controls, the saliva of PC patients demonstrated an increase of 31 bacterial species and a reduction of 25 bacterial species [80]. In another study by Sun et al. wherein the researchers analyzed the saliva samples of patients with PC (*n* = 10), benign pancreatic disease (BPD) (*n* = 17) and healthy controls (*n* = 10) using 16S rDNA high-throughput sequencing, they demonstrated high concentrations of *Fusobacterium periodonticum* and low concentrations of *Neisseria mucosa* as specific risk factors for PC, thus, indicating their role as potential microbial diagnostic markers of PC [81]. More large cohort studies are needed to determine a PC-specific microbial biomarker.

## 6. Microbiome in Cancer Therapy

Studies have shown that microbiota can significantly alter the efficacy of chemotherapy. For example, PDAC tumors enriched with Proteobacteria-γ (including *K. pneumoniae*) induced gemcitabine resistance due to the presence of a long isoform of the enzyme cytidine deaminase (CDD_L_) in the bacteria [29,82]. Interestingly, ciprofloxacin abrogated this microbiota-induced gemcitabine resistance [29]. Similarly, in pre-clinical models, gut microbiota has shown to enhance the side-effects of irinotecan-based chemotherapeutic treatment in PDAC [83,84]. An enzyme produced by mycoplasma, pyrimidine nucleoside phosphorylase, mediates the phosphorylation of uridine, 2′-deoxyuridine and thymidine, which can reduce the nucleoside-based chemotherapeutic anti-cancer outcomes [85]. In mice inoculated with *Porphyromonas gingivalis*, treatment with probiotics inhibited the expression and phosphorylation of SMAD3, thus, probably playing a role in the inhibition of mitosis and cell proliferation [86]. A xenograft tumor model treated with oral *Lactobacillus casei* induced the activation of p53-mediated apoptosis and increased the efficacy of 5-FU and cisplatin, resulting in beneficial anti-tumor outcomes [87]. However, the exact molecular basis of the probiotic-mediated anti-tumor chemotherapeutic efficacy is still unclear.

Gut microbiota is considered to enhance the antigenic repertoire and immune health, along with increasing anti-tumor responses through the inflammatory activation of tumor-infiltrating immune cells [88,89]. However, a chemical released from *Bacteroides fragilis*, polysaccharide A, induced the differentiation of naïve CD4+T cells to immunosuppressive pro-tumor regulatory CD4+T cells (Treg) [90]. Immunotherapy with checkpoint inhibitors (ICIs) has been a profound failure in PDAC [91]. While an immunosuppressive environment is seen in all solid tumors, the specific reasons behind PDAC having a distinctly poor response to ICI-based therapeutic approaches are unclear [92,93,94]. There is limited molecular understanding of the expression pattern of checkpoint inhibitors (PD1, PDL1 and CTLA4) in a PDAC tumor microenvironment. An antibiotic-treatment-mediated upregulation of PD1 expression in PDAC tumor-infiltrating immune cells resulting in positive outcomes following anti-PD1-based ICI therapy was reported in pre-clinical models [11]. In a melanoma study, the microbial composition in melanoma patients was found to be different between immunotherapy responders and non-responders. In this study, *Bifidobacterium* was associated with a better response to ICIs [10,95]. The bacterial species, *Bifidobacterium pseudolongum*, *Lactobacillus johnsonii* and *Olsenella* species have demonstrated enhanced efficacy of ICIs in four different murine cancer models through the production of inosine, which through interaction with adenosine A2A receptor induced anti-tumor CD4+T cell activation [96].

A clinical study using a co-treatment of pembrolizumab (NCT03637803) with lyophilized bacteria showed limited initial efficacy in otherwise ICI-refractory metastatic non-small cell lung cancer, renal cancer and PDAC. There is another ongoing clinical trial to evaluate the combinatorial benefit of probiotics with vancomycin and nivolumab (NCT03785210) in patients with refractory hepatocellular carcinoma and pancreatic cancers. Interestingly, two phase I clinical trials have shown that in resistant metastatic melanoma, fecal microbiota transplantation (FMT) can improve the response to ICI [97,98]. In an antibiotic-treated PC murine model, FMT caused a bacterial colonization of pancreatic tumors along with an infiltration with cytotoxic CD8+T cells [71]. Similar studies that combine FMT and immunotherapy are underway in gastrointestinal cancers, prostate cancer, non-small cell lung cancer and mesothelioma (NCT04130763, NCT04729322, NCT04521075, NCT04116775, NCT04056026, NCT03819296 and NCT04163289). In addition, the co-administration of probiotics and/or a high-fiber diet with ICI is being rigorously evaluated in renal, breast cancer, non-small cell lung and colorectal cancers (NCT03829111, NCT03775850 and NCT04909034). There is growing evidence that specific dietary changes can alter the intestinal microbiota [99], which can be associated with an altered response to immunotherapy. Despite these studies, ICI therapies in the current forms have yet to demonstrate activity in pancreatic cancer, and would likely limit the applicability of FMT to enhance ICI in this disease. Various ongoing clinical trials studying the impact (and/or characterization) of the microbiome in PC are listed in Table 2.

The application of genetically engineered microbiota in cancer therapy is an area of intense research interest. In bacteria-based cancer therapy, *Salmonella*, a facultative strain that is well-studied with a fully sequenced genome, is considered specifically attractive due to its tumor localization capability and natural toxicity [100]. Tan et al. showed that a single dose of attenuated *Salmonella typhimurium* that was bioengineered to express cytolysin A markedly inhibited the growth of murine PC xeno- and orthografts. This was accompanied by the destruction of stromal cells in PC, along with enhanced infiltration with anti-tumor immune cells [101]. Similar pre-clinical studies with *S. typhimurium* bioengineered to express collagenase and hyaluronidase demonstrated reduced collagen fibers in PC, resulting in a reduced tumor burden and proliferation [102,103]. Attenuated *Listeria monocytogenes* is another bacterium extensively studied in cancer therapy, due to its ability to efficiently activate TLR-mediated innate immune responses and antigen presentation, resulting in CD8+T-cell-mediated anti-tumor responses [104,105,106]. Bioengineered *Listeria monocytogenes* designed to express mesothelin, a PC-associated tumor antigen, induced the efficient activation of cytotoxic CD8+T cell responses, inducing PC tumor regression [107,108]. Unfortunately, a human trial based on this approach was disappointing, as it was not better than standard conventional chemotherapy [109,110,111]. However, future studies combining standard chemo-/immunotherapy with engineered microbiota could provide better outcomes.

## 7. Conclusions

The role of microbiota on carcinogenesis is an emerging area of research. Several, apparently contradictory, observations have only pointed to a need for more thorough and standardized future studies to analyze this problem, which include robust sample collections, study size and defining the normal microbiota among various demographics and geographical distributions. This understanding could lead to future applications of microbiome-based diagnostic/prognostic biomarkers and therapeutic interventions with probiotics, FMT and/or antibiotic co-treatment. Further, more detailed molecular studies are needed to uncover the functional impact of the microbiome in pancreatic cancer. Such immunological and biochemical studies would provide deeper insights into microbiome-mediated pancreatic carcinogenesis to enable the development of novel therapies to reduce the mortality and quality of life of pancreatic cancer patients.

## Figures and Tables

**Table 1:** Microbial taxa with propensity to cause pancreatic cancer.

	Microbial Taxa	Impact of PC Tumorigenesis	Model/Methodology
**Oral Microbiota**	Leptotrichia	Enhances [18,20]; Inhibits [19]	Ref#[18,19,20] Human—16S rRNA gene sequencing
	Fusobacterium	Enhances [21,22,23]	Ref#[21] Human—TaqMan gene expression Assay for Fusobacterium (RT-qPCR); Ref#[22] Human—16S rRNA gene sequencing; Ref#[23] Human—ELISA and RT-qPCR on saliva
	Haemophilus	Inhibits [18]	Ref#[18] Human—16S rRNA gene sequencing
	Porphyromonas gingivalis	Enhances [19,24]	Ref#[19] Human—16S rRNA gene sequencing; Ref#[24] Human—serology/plasma antibodies
	Aggregatibacter	Enhances [19]	Ref#[19] Human—16S rRNA gene sequencing
**Gut Microbiota/Mycobiota**	Helicobacter pylori	Enhances [25,26,27]	Ref#[25,26,27] Human—meta-analysis
	Proteobacteria	Inhibits [28]	Ref#[28] Human—meta-analysis RNA-sequencing data from The Cancer Genome Atlas (TCGA)
	Bifidobacterium	Enhances [11]	Ref#[11] Human—16S rRNA gene sequencing
	Ascomycota (fungi)	Enhances [13]	Ref#[13] Human- MiSeq, alpha and beta diversity indices (Chao1, Shannon and Simpson indices)
	Ascomycota (fungi)	Enhances [13]	Ref#[13] Human—MiSeq, alpha and beta diversity indices (Chao1, Shannon and Simpson indices)
**Intratumoral Microbiota/Mycobiota**	Proteobacteria	Enhances [11]	Ref#[11] Human—16S rRNA gene sequencing
	Enterobacteriaceae	Enhances [29]	Ref#[29] Human—rRNA fluorescence in situ hybridization
	Pseudomonadaceae	Enhances [11,29]	Ref#[11] Human—16S rRNA gene sequencing
	Bacteroidetes	Enhances [16]	Ref#[16] Human—shotgun metagenomic and 16S rRNA amplicon sequencing
	Malassezia (fungi)	Enhances [13]	Ref#[13] Human—MiSeq, alpha and beta diversity indices (Chao1, Shannon and Simpson indices);

**Table 2:** Clinical trials to study the microbiota associated with pancreatic cancer.

Intervention	Identifier#	Title	Tumor Type	Status
16S rRNA gene sequencing assay	NCT03302637	Oral Microbiome and Pancreatic Cancer	Pancreatic Cancer	Completed
Diagnostic test: microbiome evaluation	NCT04274972	The Microbiome of Pancreatic Cancer: „PANDEMIC” Study (PANDEMIC)	Pancreas Cancer	Unknown
Diagnostic test: dental plaque sampling (qPCR)	NCT04993846	Pancreatic Cancer and Oral Microbiome	Pancreas Cancer	Recruiting
Other: oral and rectal swabs for microbiome sequencing	NCT04922515	Pancreatic Ductal Adenocarcinoma—Microbiome as Predictor of Subtypes (PDA-MAPS)	Pancreatic Cancer	Recruiting
Phase 4 Drug: pembrolizumab, ciprofloxacin and metronidazole	NCT03891979	Gut Microbiome Modulation to Enable Efficacy of Checkpoint-based Immunotherapy in Pancreatic Adenocarcinoma	Pancreatic Cancer	Withdrawn
Drug: immunotherapy and chemotherapeutic agent microbiome analysis	NCT04638751	ARGONAUT: Stool and Blood Sample Bank for Cancer Patients	Non-Small Cell Lung Cancer, Colorectal Cancer, Triple Negative Breast Cancer, Pancreas Cancer	Recruiting
Microbiome analysis	NCT04189393	Microbiome Analysis in esoPhageal, PancreatIc and Colorectal CaNcer Patients Undergoing Gastrointestinal Surgery (MA-PPING)	Gastrointestinal Cancer, Colorectal Cancer, Pancreatic Cancer, Esophageal Cancer	Unknown
Microbiota analysis	NCT04931069	Correlation between Complications after Pancreaticoduodenectomy and Microbiota (COMPAMIC)	Pancreatic Cancer	Recruiting
Phase 1 Drug: MRx0518 Radiation: hypofractionated pre-operative radiation	NCT04193904	A Study of Live Biotherapeutic Product MRx0518 With Hypofractionated Radiation Therapy in Resectable Pancreatic Cancer	Pancreatic Cancer	Recruiting
phase 1 and 2 Drug: durvalumab Radiation: stereotactic ablative body radiotherapy (SABR) Microbiome analysis	NCT03245541	Radiation Therapy in Combination With Durvalumab for People With Pancreatic Cancer	Pancreatic Adenocarcinoma	Recruiting
Observational study only microbiome analysis	NCT04476082	Nutrition in Gastrointestinal Tumors (NutriGIT)	Pancreatic Cancer, Oesophageal Cancer, Colon Cancer, Liver Cancer, Rectal Cancer, Bile Duct Cancer	Recruiting
